# Remission of Type II Diabetes Mellitus after Duodenal Switch: the Contribution of Common Channel Length

**DOI:** 10.1007/s11695-023-06870-2

**Published:** 2023-10-10

**Authors:** Lindsey S. Sharp, William T. Sharp, Peter Ng

**Affiliations:** UNC Rex Healthcare, Rex Bariatric Specialists, 4207 Lake Boone Trail, Suite 210, Raleigh, NC 27607 USA

**Keywords:** Diabetes remission, Duodenal switch, Biliopancreatic diversion, Single anastomosis duodenoileostomy with sleeve gastrectomy

## Abstract

**Introduction:**

The role of the common channel length in duodenal switch (DS) on remission of type II diabetes mellitus (DM), when stratifying patients based on diabetes severity, is not well understood.

**Methods:**

We retrospectively reviewed 341 consecutive patients with DM undergoing DS with one of three different common channel (CC) lengths (100 cm, 150 cm, and 200 cm), each with a fixed 300 cm alimentary limb (AL). Patients were stratified by insulin dependence (IDDM) versus non-insulin dependent diabetes (NIDDM). Data was collected at one year and at the last available follow-up.

**Results:**

The NIDDM group had a similar average HbA1c at last follow-up for each of the CC lengths. However, the IDDM group had lower average HbA1c with shorter CC lengths (100 cm = 5.4%, 150 cm = 6%, 200 cm = 6.4%, *p* < 0.05). Shorter CC lengths resulted in a greater proportion of patients achieving remission in the IDDM group (66%, 50%, 32% in the 100 cm, 150 cm, and 200 cm CC, respectively, *p* < 0.01). Improvements in HbA1c were independent of weight loss and average DiaRem scores were similar between CC lengths. Rates of nutritional deficiencies were higher in shorter common channel lengths. Revision for malnutrition was similar between common channel lengths (100 cm group: 3.7%; 150 cm group: 1.8%; 200 cm group: 0%, *p* = NS).

**Conclusions:**

When the AL is fixed, shortening CC lengths results in improved glycemic control and remission of DM in patients with the need for insulin preoperatively. Milder forms of DM are treated well with any of the CC lengths.

**Graphical Abstract:**

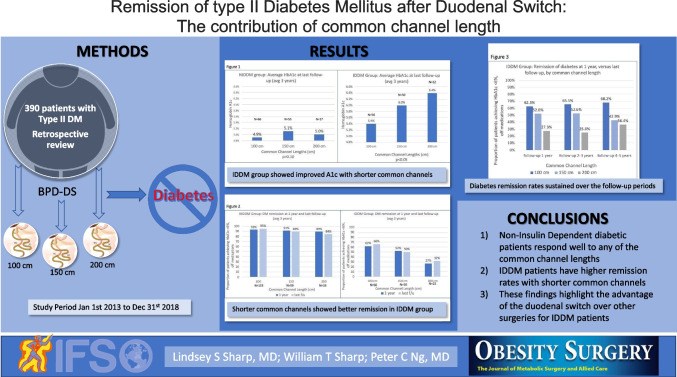

## Introduction

According to the Centers for Disease Control and Prevention, 30 million Americans suffer from Type 2 diabetes mellitus and 84 million, pre-diabetes (https://www.cdc.gov/chronicdisease/resources/publications/factsheets/diabetes-prediabetes.htm). Predictive models project that T2DM will grow to affect nearly 55 million Americans and pre-diabetes, 108 million by 2030 [1]. The obesity epidemic closely parallels this increase in Type 2 diabetes mellitus and metabolic surgery offers the most effective treatment for type II DM in patients with obesity [2, 3].

Several scoring models exist to predict diabetes remission after bariatric surgery. Designed around gastric bypass, the DiaRem, Advanced DiaRem, ABCD, and the IMS prediction models analyze preoperative variables and laboratory values to estimate potential for remission of T2DM after surgery [4–8]. Limited efforts attempt to expand their predict outcomes to alternative techniques, such as sleeve gastrectomy. However, the current tools lack clinical validation in malabsorptive procedures, such as biliopancreatic diversion with duodenal switch (DS), ironically, the metabolic operation considered the most effective tool for T2DM.

Bariatric operations vary in design and technical construction, changing metabolic and malabsorptive effects. In Roux-en-Y gastric bypass (RYGB) operations, the roux, biliopancreatic, and common channel limb lengths prove widely variable. Surgeons routinely adjust lengths to improve weight loss and diabetes remission [9–11]. In the original biliopancreatic diversion with duodenal switch (BPD-DS), the total alimentary and common channel limb lengths measured 250 cm and 50–75 cm, respectively, evolving in more modern US practice to longer 300 cm total alimentary and 100–150 cm common channel limb lengths [12, 13]. The newest iteration, the single anastomosis duodenal switch (SADI-S), uses a 250–300 cm alimentary limb. [14, 15].

Conceptually, bariatric surgery is a continuum of operations proceeding from those gastric focused operations (sleeve gastrectomy, vertical banded gastroplasty) to operations that employ varying degrees of intestinal bypass with biliopancreatic diversion (RYGB, one anastomosis gastric bypass (OAGB), SADI-S, BPD-DS). Likewise, diabetes is a continuum with increasing levels of disease severity. The observed role of the intestinal bypass and, more specifically, longer biliopancreatic limb bypass seems to favorably influence diabetes remission.

This study evaluates the role of common channel lengths (100, 150, and 200 cm) in BPD-DS for improving glycemic control in bariatric patients with T2DM and further delineates differences in remission of T2DM, within the framework of a modified Diarem predictive model.

## Research Design and Methods

Retrospectively, the charts of all patients who underwent a BPD-DS between Jan 1^st^ 2013 and December 31^st^, 2018 were reviewed for medical history of T2DM, elevated HbA1c above 6.5%, or preoperative use of anti-diabetic medications. The review of 1224 patients identified 430 patients with T2DM. Of the 430 patients with T2DM, 416 had complete data to calculate a Diarem Score and predict diabetes remission postoperatively. Of these, 341 patients had common channel lengths of either 100 cm, 150 cm, or 200 cm with a fixed 300 cm total alimentary limb length.

The duodenal switch operations were performed with a loose sleeve gastrectomy over a 40 F bougie. The alimentary limb measured 300 cm in all patients. Patients received a common channel length of either 100 cm, 150 cm, 200 cm based on Body Mass Index (BMI). Patients with a BMI ≥ 50 received a 100 cm common channel, BMI 40–49 received a 150 cm common channel, and patients with a BMI < 40 received a 200 cm common channel. This algorithm represented our standard approach to duodenal switch procedures and all operations were performed laparoscopically.

Data was collected on preoperative variables to calculate the DiaRem Score: age, preop HbA1c, use of pre-operative insulin, gender, BMI, and common channel length. Postoperative variables were also collected at one year and last follow-up for percent total weight loss (%TWL), and the use of diabetic medication to determine remission of T2DM.

### DiaRem Score

The DiaRem Score was calculated using the method reported by Still et al. Patient age at the time of surgery determined their age score (Age < 40, score = 0; Age 40–49, score = 1; Age 50–59, score = 2; and Age 60 + , score = 3). The preoperative HbA1c closest to the time of surgery determined the A1c score (HbA1c < 6.5%, score = 0; HbA1c 6.5–6.9%, score = 2; HbA1c 7.0–8.9%, score 4; HbA1c 9.0% + , score = 6). The ISA + Sulfonylurea score was determined based on the use of an insulin sensitizing agent (ISA) (thiazolidinediones) and a sulfonylurea concurrently (No ISA + Sulfonylurea, score = 0; Yes ISA + Sulfonylurea, score = 3). The use of insulin added a score of 10 and the absence of insulin use added 0 to the DiaRem Score [4].

### Definition of Remission of Type II DM

T2DM was defined as a preoperative HbA1c of 6.5% or greater, a history of T2DM in the medical record, or the use of antidiabetic medications. Remission was defined as a HbA1c < 6.0% (or fasting blood glucose < 100 mg/dl) for at least one year duration without the use of anti-diabetic medications.

### Statistical Analysis

SAS JMP version 16 statistical software was used for statistical analysis. Pearson’s Chi square test was used to analyze differences between categorical variables. Two tailed t-tests were used to analyze distribution means.

## Results

The cohort consisted of 29.3% male and 71.7% female patients. The cohort demonstrated class III obesity with an average BMI of 49. The average age was 50 years old. The average preoperative HbA1c was 7.6% with 41% of patients requiring insulin prior to surgery. Overall, 248 (76.3%) out of the 325 patients had remission of T2 DM (median follow-up 3 years, range: 1–7.5 years).

Preoperative variables are shown in Tables 1 and 2. In patients not on insulin preoperatively (NIDDM group), there was a difference in preoperative BMI, and age between the common channel lengths. Patients in the 100 cm group trended slightly younger. In the patients on insulin preoperatively (IDDM group), there was only a difference in preop BMI between the common channel lengths and no statistically significant difference in age, HbA1c, or sex. There was minimal difference in pre-operative DiaRem score between the common channel lengths for the NIDDM group (average DiaRem 3 for 100 cm, 4 for 150 cm, 3 for 200 cm). There was no difference in pre-operative DiaRem score in the IDDM group (average DiaRem 16 for each common channel length).

**Table 1:** Preoperative variables for NIDDM patients

Common channel length	100 cm	150 cm	200 cm	*P*-value
Preop BMI	53	46	39	*p* < 0.01
Female sex	68%	78%	79	0.31
Age	46	52	48	100–150 *p* = 0.0004 100–200 *p* = 0.51 150–200 *p* = 0.12
Preop HbA1c	7%	6.8%	6.4%	100–150 *p* = 0.22 100–200 *p* = 0.06 150–200 *p* = 0.17
Diarem score	3	4	3	100–150 *p* = 0.07 100–200 *p* = 0.18 150–200 *p* = 0.02

**Table 2:** Preoperative variables for IDDM patients

Common channel length	100 cm	150 cm	200 cm	*P*-value
Number of patients	188	111	42	*p* < 0.0001
Preop BMI	52	44	39	
Female sex	63%	69%	83%	0.22
Age	51	54	55	100–150 *p* = 0.18 100–200 *p* = 0.15 150–200 *p* = 0.69
Preop HbA1c	8.8%	8.7%	8.5%	100–150 *p* = 0.87 100–200 *p* = 0.51 150–200 *p* = 0.60
Diarem score	16	16	16	100–150 *p* = 0.33 100–200 *p* = 0.40 150–200 *p* = 0.93

Nutritional data is presented in Tables 3 and 4. Vitamin deficiency rates for vitamins A and D were higher in the shorter common channels, both preoperatively and at 1 year and last follow-up. Rates of hypoalbuminemia were also higher in the shorter common channel group at 1 year and last follow-up. Surgical revision for malnutrition was 3.7%, 1.8%, and 0% for the 100, 150, and 200 cm common channel groups, respectively (*p* = 0.62). Rates of secondary hyperparathyroidism were similar between common channel groups. There was no difference in rates of hypocalcemia between common channel groups. Rates of low copper and zinc levels also were higher in the shorter common channel lengths. Anemia rates were similar between common channel lengths at last follow-up, but increased within each common channel length overtime. Similarly, ferritin levels tended to fall over time, although there was no statistically significant difference in rates of low ferritin levels between common channel lengths.

**Table 3:** Nutritional data by common channel length

Vitamin D Preoperative				
Common channel	100 cm	150 cm	200 cm	
Insufficiency (20–30 ng/ml)	26%	35%	40%	
Deficiency (< 20 ng/ml)	48%	24%	13%	
Total	74%	59%	53%	*p* = 0.008
Vitamin D 12 months postoperative				
Common channel	100 cm	150 cm	200 cm	
Insufficiency (20–30 ng/ml)	26%	18%	8%	*p* = 0.03
Deficiency (< 20 ng/ml)	23%	5%	8%	*p* = 0.0003
Total	49%	23%	15%	*p* < 0.000001
Vitamin D Last follow-up				
Common Channel	100 cm	150 cm	200 cm	
Insufficiency (20–30 ng/ml)	31%	26%	17%	*p* = 0.21
Deficiency (< 20 ng/ml)	28%	14%	9%	*p* = 0.007
Total	61%	40%	26%	*p* = 0.0001
Albumin Preoperative				
Common channel	100 cm	150 cm	200 cm	
Mild (3.4–3.0 g/dL)	16.8%	18.3%	4.8%	
Moderate (2.9–2.5 g/dL)	2.6%	0.0%	4.8%	
Severe (< 2.5 g/dL)	0.6%	0.0%	2.4%	
Total	20.0%	18.3%	11.9%	*p* = 0.48
Albumin 12 months posteroperative				
Common channel	100 cm	150 cm	200 cm	
Mild (3.4–3.0 g/dL)	17.1%	11.4%	7.7%	*p* = 0.20
Moderate (2.9–2.5 g/dL)	8.2%	1.0%	2.6%	*p* = 0.02
Severe (< 2.5 g/dL)	2.9%	1.0%	0.0%	*p* = 0.55
Total	28.2%	13.3%	10.3%	*p* = 0.003
Albumin Last follow-up				
Common channel	100 cm	150 cm	200 cm	
Mild (3.4–3.0 g/dL)	20.0%	8.0%	18.0%	*p* = 0.03
Moderate (2.9–2.5 g/dL)	5.0%	4.0%	0.0%	*p* = 0.73
Severe (< 2.5 g/dL)	3.0%	4.0%	0.0%	*p* = 0.82
Total	29.0%	17.0%	18.0%	*p* = 0.045

**Table 4:** Nutritional data by common channel length

Vitamin A 12 months postoperative					
Common channel	100 cm	150 cm	200 cm		
Mild (31–21 mcg/dl)	24.7%	10.6%	5.6%	*p* = 0.003	
Moderate (20–10 mcg/dl)	10.0%	4.3%	2.8%	*p* = 0.13	
Severe (< 10 mcg/dl)	2.0%	1.1%	0.0%	*p* = 0.78	
Total	36.70%	16%	8.30%	*p* = 0.0001	
Vitamin A Last follow-up					
Common channel	100 cm	150 cm	200 cm		
Mild (31–21 mcg/dl)	17.4%	14.7%	5.9%	*p* = 0.23	
Moderate (20–10 mcg/dl)	6.0%	2.7%	0.0%	*p* = 0.46	
Severe (< 10 mcg/dl)	6.0%	0.0%	0.0%	*p* = 0.23	
Total	29.5%	17.3%	5.9%	*p* = 0.00495	
PTH level 12 months					
Common channel	100 cm	150 cm	200 cm		
> 80 pg/ml	34%	22%	19%	*p* = 0.0452	
> 100 pg/ml	16%	13%	8%	*p* = 0.46	
PTH level Last follow-up					
Common channel	100 cm	150 cm	200 cm		
> 80 pg/ml	55%	52%	41%	*p* = 0.35	
> 100 pg/ml	42%	33%	29%	*p* = 0.26	
Calcium (< 8.4 mg/dL)	100 cm	150 cm	200 cm		
Preoperative	0.7%	0.0%	0.0%	*p* = 0.60	
12 months	1.8%	2.9%	0.0%	*p* = 0.83	
Last follow-up	6.0%	3.2%	0.0%	*p* = 0.464	
Vitamin B-12 (< 211 pg/mL)	100 cm	150 cm	200 cm		
Preoperative	2.9%	2.3%	0.0%	*p* = 0.98	
12 months	1.2%	0.0%	0.0%	*p* = 0.78	
Last follow-up	0.7%	1.2%	0.0%	*p* = 0.587	
Copper (< 0.73 mcg/dL)	100 cm	150 cm	200 cm		
12 months	10.0%	1.0%	3.0%	*p* = 0.011	
Last follow-up	10.0%	7.0%	0.0%	*p* = 0.30	
Zinc (< 0.6 mcg/dL)	100 cm	150 cm	200 cm		
12 months	35%	19%	11%	*p* = 0.00146	
Last follow-up	40%	20%	18%	*p* = 0.0020	
Hemoglobin (< 11.3 g/dL)	100 cm	150 cm	200 cm		
Preoperative	10.2%	7.6%	5.0%	*p* = 0.507	
12 months	20.8%	11.5%	7.7%	*p* = 0.039	
Last follow-up	27.1%	23.5%	18.4%	*p* = 0.51	
	*p *= 0.0003	*p *= 0.003	*p* = 0.12		
Ferritin (< 10.5 ng/ml)	100 cm	150 cm	200 cm		
Preoperative	4.4%	2.1%	2.8%	*p* = 0.62	
12 months	4.3%	10.0%	7.7%	*p* = 0.19	
Last follow-up	14.8%	12.2%	19.4%	*p* = 0.59	
	*p *= 0.0006	*p* = 0.03	*p* = 0.052		

**Fig. 1:**
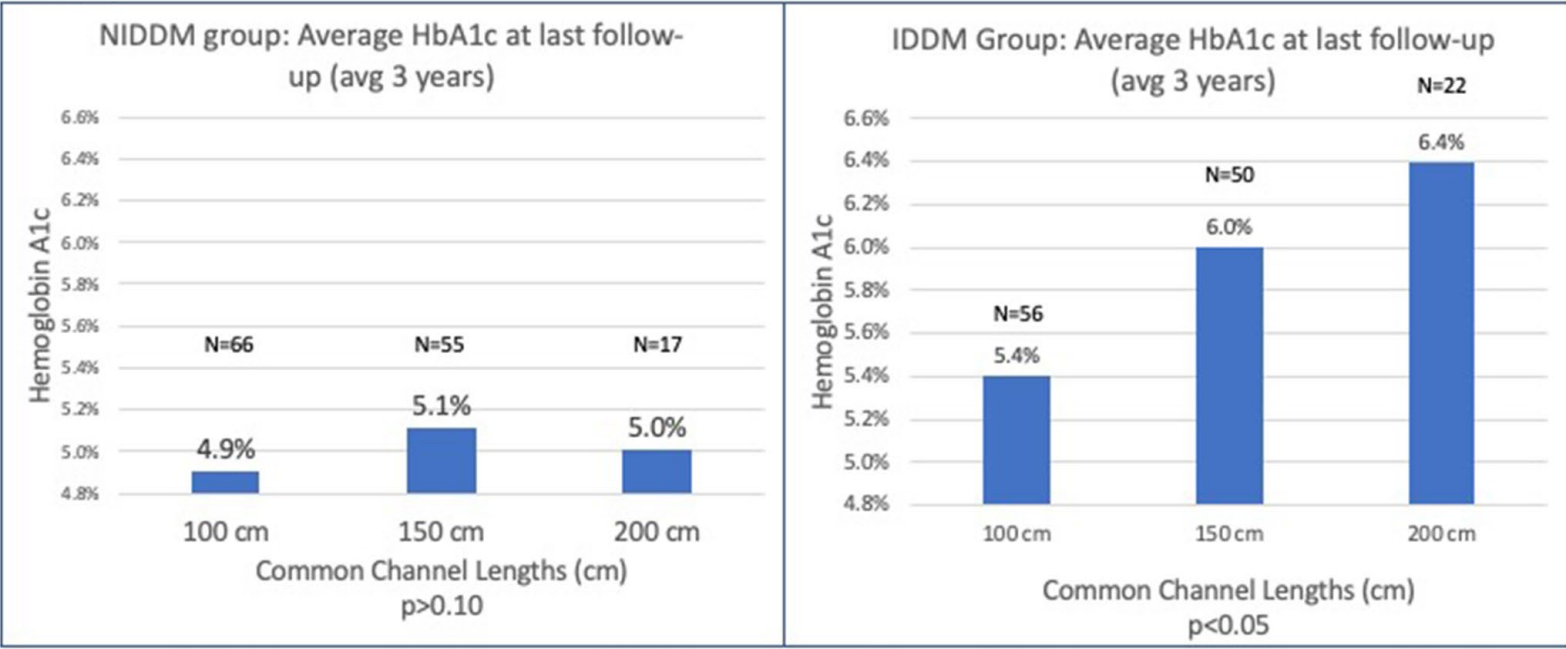
No statistically significant difference between common channel lengths found in HbA1c achieved at last follow-up in the NIDDM group. Conversely, a statistically significant difference was seen in the HbA1c achieved at last follow-up in the IDDM group

For the NIDDM group, 93% (*n* = 176/189) demonstrated diabetes remission at one year and 91% (*n* = 183/201) diabetes remission at last follow-up (Fig. 1). For IDDM group patients, 53% (72/136) demonstrated remission at one year, and 55% (*n* = 77/140) remission at last follow up.

Glycemic improvement was compared between the common channel lengths and stratified by IDDM or NIDDM groups preoperatively. In the NIDDM group, the average HbA1c achieved at last follow up (median 3 years, range 1–7.5 years) was similar between the common channel lengths (HbA1c = 4.9%, 5.1%, 5.0% for 100 cm, 150 cm, and 200 cm, respectively, p = NS). In contrast, the average HbA1c in the IDDM group at last follow-up increased based on limb length. A statistically significant trend related to longer common channel lengths was identified (5.4%, 6.0%, and 6.4% for the 100 cm, 150 cm, and 200 cm common channel groups, respectively, 100–150 cm *p* = 0.002, 100–200 cm *p* < 0.001, 150–200 cm *p* = 0.048) (Fig. 1).

**Fig. 2:**
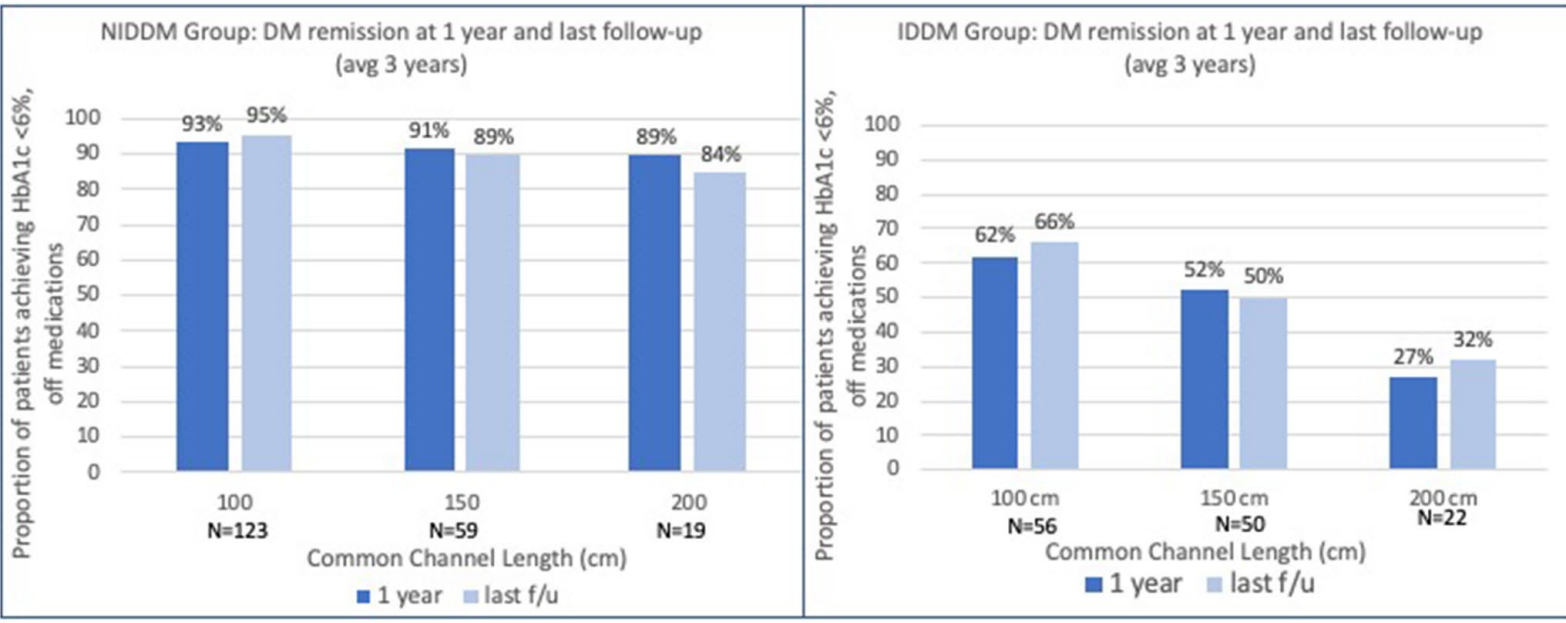
No statistically significant rate of remission of DM in the NIDDM group at 1 year or last follow-up (*p* = 0.45 and *p* = 0.43, respectively). Significant difference in rate of DM remission in the shorter common channels at 1 year and last follow-up (*p* = 0.02 and *p* < 0.01, respectively)

In the IDDM group, the proportion of patients achieving a HBA1c < 6% off medications at 1 year and last follow-up are presented in Fig. 2. Remission rates were superior in the shorter common channels at one year (62%, 52%, 27% in the 100 cm, 150 cm, and 200 cm CC, respectively, p = 0.02) and last follow-up (66%, 50%, 32% in the 100 cm, 150 cm, and 200 cm CC, respectively) (*p* < 0.01). In the NIDDM group, remission rates were similar among the three common channel lengths at one year (93%, 91%, 89% in the 100 cm, 150 cm, and 200 cm CC, respectively) (*p* = 0.45) and last follow-up (95%, 89%, 84% in the 100 cm, 150 cm, and 200 cm CC, respectively) (*p* = 0.43).

**Fig. 3:**
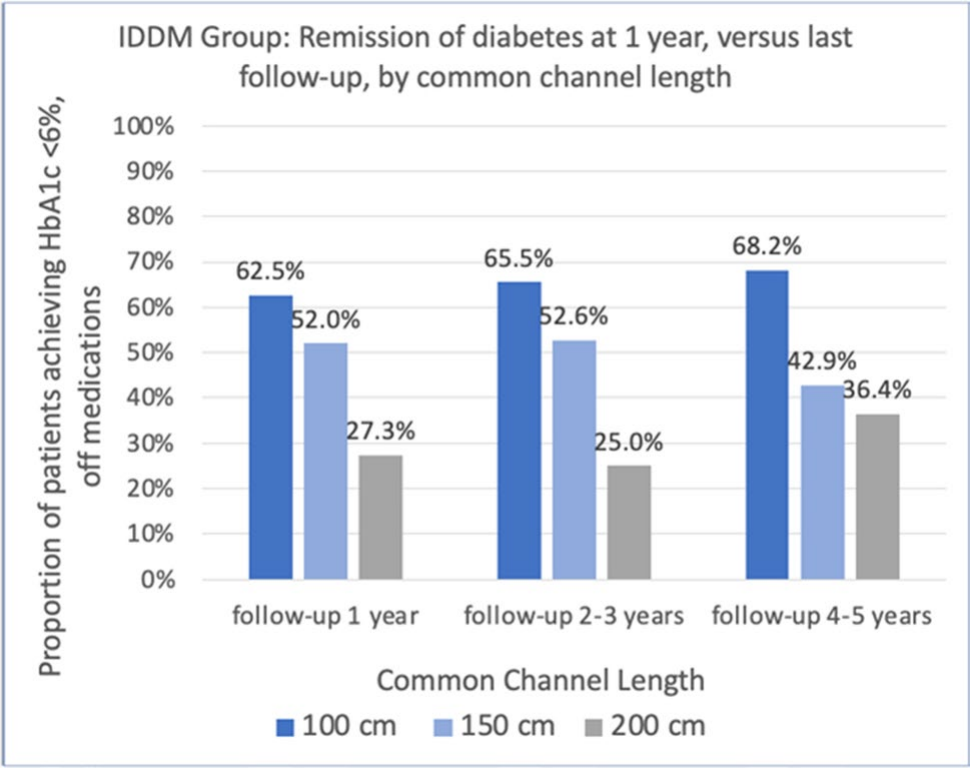
Remission of DM at 1 year and at last follow up showed significant difference in remission between common channel lengths at each time period, but no difference in remission over the last follow-up within a given common channel length. (*p*-values listed in Table 3)

For the IDDM group, remission of T2DM was sustained over the 5 year period. The DM remission rate in the 100 cm common channel group was 62.5%, 65.5%, and 68.2% at 1 year, 2–3 years, and 4–5 years postoperatively, respectively (p = NS). Likewise, for the 150 cm group remission rates were 52%, 52.6%, and 42.9% at 1 year, 2–3 years, and 4–5 years postoperatively, respectively (p = NS). The 200 cm common channel length remission rates were poor throughout the study period; 27%, 25%, and 36.4% at 1 year, 2–3 years, and 4–5 years postoperatively, respectively (p = NS) (Fig. 3 and Table 5).

**Table 5:** Remission of DM over time. No difference within a given common channel length

Common Channel	1 year follow up	2–3 years last follow up	4–5 years last follow up	*P*-value
100 cm	62.5%	65.5%	68.2%	*p* = 0.59
150 cm	52.0%	52.6%	42.9%	*p* = 0.15
200 cm	27.3%	25.0%	36.4%	*p* = 0.59
	*p* = 0.006	*p* = 0.026	*p* = 0.028	

Significant difference between common channels for each follow up period

As a cohort, DiaRem score accurately classified likelihood of diabetes remission. The T2DM remission rate for DiaRem scores were as follows: score 0–4 = 99%; score 5–8 = 93%; score 9–13 = 80%; score 14–18 = 59%, score 19–22 = 25% (*p* < 0.0001). Only 1% of the patients were on an ISA + Sulfonylurea.

## Discussion

In the ideal world, metabolic surgery would provide an operation with 100% efficacy with 0% risk. While far from the ideal, BPD-DS offers many important and valuable benefits, specifically for T2DM. Understanding the nutritional and metabolic impact of malabsorptive procedures, we have attempted to balance the risk of nutritional and gastrointestinal complications and optimize metabolic outcomes by tailoring common channel lengths in BPD-DS. We examined the impact of a BMI based strategy on T2DM remission and stratified patients based on diabetes severity. We have demonstrated that increasing the common channel length over a fixed total alimentary limb length decreases the likelihood of T2DM remission, specifically for patients with severe disease as indicated by higher DiaRem scores or requiring insulin preoperatively. In contrast, non-severe T2DM patients showed similar diabetes remission across all common channel lengths.

The concept of common channel length impacting the insulin-glucose cycle is well studied. Pereira et al. have evaluated the effects of common channel length on circulating gut hormones after a mixed-meal tolerance test [16]. They compared the insulin, glucose, and other gut hormone responses in non-diabetic patients subjected to either a 100 cm common channel in Duodenal Switch versus 300 cm common channel in SADI-S [16]. Interestingly, the glucose, insulin, and glucagon responses were uniformly lower in the BPD-DS group compared to the SADI group. The glucose-dependent insulinotropic polypeptide (GIP) and glucagon-like peptide (GLP-1) responses were also lowest in the BPD-DS compared to SADI. The response of these circulating gut hormones suggests there may be a dose response effect to the shortening of the common channel. The authors offered the following conclusion: “The post-prandial response observed after BPD-DS and SADI-S may suggest that the later procedure could be advantageous in patients with insulin resistance, pre-diabetes, and diabetes in earlier stages of the disease with reasonable pancreatic reserve, while BPD-DS is likely to perform better in those patients with more advanced stages of the disease” [16].

The distribution of glucose transporters in the gut, such as sodium-glucose linked transporters (SGLT-1) and facilitative glucose transporters (Glut2) may contribute to the differences seen in common channel length. SGLT-1 is located on the brush border membrane of intestinal cells and requires 2 sodium molecules and ATP to transport 1 glucose molecule into the intestinal cells. Once inside the enterocytes, Glut-2 transports glucose across the basolateral membrane into the extracellular fluid [17]. Biliopancreatic fluid carries the highest concentration of sodium of any other body fluid [18]. Even over the same alimentary limb length, a shorter common channel results in a shorter length of intestine where sodium-rich biliopancreatic fluid is available for glucose absorption via SGLT-1 glucose transporters, thereby reducing overall glucose absorption and the need for a high insulin response. A significant portion of sodium excretion also occurs in the stomach and gastric volume reduction via the sleeve gastrectomy may also contribute to the improved postprandial glycemic response [19].

GLUT5 is a facilitative fructose transporter on the apical membrane of intestinal cells [20]. When concentrations of fructose are high in the intestinal lumen, fructose if transported into the intestinal cells via GLUT5 and then across the basolateral membrane via GLUT2 [20]. The distribution of GLUT5 transporters in mainly in the proximal small intestine, namely the duodenum and proximal jejunum [20]. There are far fewer GLUT5 transporters in the ileum. GLUT5 transporters are upregulated in the proximal intestine in response to high fructose diets and in type II DM [20]. Following RYGB, GLUT5 is upregulated in the roux-limb which may result in increased caloric absorption overtime and may contribute to weight regain and recidivism of type II DM.

Enteroendocrine cells express glucose transporters, which allow them to respond to high intraluminal glucose states, similar to K cells which release GIP and L cells which release GLP-1, GLP-2, and PYY. A reduction in exposure to sodium rich biliopancreatic fluid may, in part, explain the lower levels of GIP and GLP-1 following shorter common channel procedures. In humans with T2DM, upregulated concentrations of SGLT-1 and Glut 2 have been found at levels 4 × greater than healthy individuals [17]. Although speculative, decreasing the absorption of glucose via these transporters may offer a more profound anti-glycemic effect in patients with more severe diabetes.

Scoring systems such as DiaRem offer important clinical value, allowing researchers/surgeons to predict T2DM remission based on disease severity and, equally, compare the efficacy of both current techniques (e.g. VSG, RYGB, SADI, and DS) and new approaches (single anastomosis sleeve to ileum bypass, sleeve with bipartition, endoscopic procedures). Most importantly, surgeons and patients may choose the most effective procedure for their obesity and comorbidity. For example, based on the original DiaRem study by Still et al., RYGB offered to a patient with a DiaRem score in the 13–17 range realized a 5–16% chance of diabetes remission., Compare this to our study cohort, BDP-DS with the similar DiaRem score range (14–18) realized a 52% remission rate [4]. Additionally, in Sanchez-Pernaute’s study on SADI-S in IDDM patients, they report a remission rate of 47% at one year, and 54–56% at 2–3 years, and 38–47% at 4–5 years, compared to our data which showed a 63%, 66%, and 68% remission over similar time periods [14]. This highlights the differences in diabetes remission between procedures and unmasks the differential effects of diabetes severity on short and long term outcomes.

The price to be paid for improved diabetes remission with shorter common channels are higher rates of fat-soluble vitamin deficiencies and consequently the need for a more aggressive vitamin supplementation regimen. In our practice, we currently prescribe dry vitamin D3 (cholecalciferol) at doses starting at 35,000 IU daily. We will titrate the dose higher to achieve a serum total vitamin D level of 60–80 ng/ml to help normalize PTH levels. The likelihood of hyperparathyroidism is 6 × higher in patients who fail to achieve this vitamin D target [21]. The target vitamin D level of 60–80 ng/ml are much more easily achieved with lower daily doses of vitamin D supplementation in our SADI-S patients. Hypoabluminemia is also more common in the 100 cm common channel DS, but tends to be mild. The revision rate for malnutrition for 100 cm common channel DS (3.7%) is similar to those published elsewhere for DS and decreases with increased common channel length (although not achieving statistical significance in this study).

There are limitations and potential biases in our study. Patients were not randomized between the treatment groups (100 vs. 150 vs. 200 cm common channels), potentially creating selection bias. We believe this factor is mitigated by multivariate analysis and because preoperative BMI was not a predictive factor for diabetes remission. Additionally, we were unable to account for duration of disease for diabetes as this data was not collected. Duration of disease may have further defined predictive outcomes as disease duration is known to impact the potential for T2DM remission.

## Conclusion

In patients with severe diabetes, as indicated by the need for insulin preoperatively, better glycemic control and higher remission rates were seen with shorter common channel lengths in duodenal switch. Predicting the response of diabetes to bariatric operations is important, not only for setting patient expectations, but also for reducing risk. While insulin use preoperatively is a rough predictor of diabetes severity, other tools such as the DiaRem score may be better at determining diabetes disease severity. The BPD-DS based DiaRem predictive model guides surgeon and patient alike in choosing a metabolic operation, understanding and selecting the common channel length, and predicting the opportunity for diabetes remission, all in the larger context of T2DM severity, weight loss goals, age, comorbidities, nutritional and perioperative risks.

## Data Availability

Data supporting this study may be available upon request from the corresponding author persuant to University of North Carolina data sharing policies.
